# *PARP1* Gene Polymorphisms and the Prognosis of Esophageal Cancer Patients from Cixian High-Incidence Region in Northern China

**DOI:** 10.31557/APJCP.2020.21.10.2987

**Published:** 2020-10

**Authors:** Rong-Miao Zhou, Yan Li, Na Wang, Chao-Xu Niu, Xi Huang, Shi-Ru Cao, Xiang-Ran Huo

**Affiliations:** 1 *Hebei Provincial Cancer Institute, The Fourth Hospital of Hebei Medical University, Hebei Province, China. *; 2 *Department of Surgery, Shijiazhuang Ping’an Hospital, Hebei Province, China.*

**Keywords:** Poly(ADP-ribose) polymerase 1, polymorphism, prognosis, survival, esophageal squamous cell carcinoma

## Abstract

**Objective:**

Poly (ADP-ribose) polymerase 1 (PARP1), as a key enzyme in the base excision repair pathway, plays a crucial role in tumorigenesis and progression. This study aimed to assess whether polymorphisms of *PARP1* gene could be used as predictive biomarkers for the survival of esophageal squamous cell carcinoma (ESCC) patients from Cixian high-incidence region in northern China.

**Methods:**

In 203 ESCC patients with survival information, *PARP1 *rs1136410 T/C and rs8679 T/C single nucleotide polymorphisms (SNPs) were genotyped by polymerase chain reaction ligase detection reaction (PCR-LDR) method. All statistical analyses were performed using the SPSS ver. 22.0 software package (SPSS, Chicago, IL, USA).

**Results:**

The mean age ± standard deviation of the ESCC patients was 60.4 ± 7.9 years. There was no significant relation of sex, age, smoking status and upper gastrointestinal cancer family history with the survival time of the ESCC patients. The mean survival time of rs1136410 T/T, T/C and C/C genotype carriers were 43.3, 42.3 and 46.6 months, respectively. The rs1136410 was not associated with the survival time of the ESCC patients. For rs8679, the mean survival time of T/T genotype carriers was 43.7 months, which was not significantly different from that of the patients with T/C genotype (42.1 months).

**Conclusion:**

In Cixian high-incidence region from northern China, rs1136410 and rs8679 SNPs might not be used to predict survival of ESCC patients. There is a need to explore whether other SNPs of *PARP1* gene have an effect on prognosis of ESCC patients.

## Introduction

DNA repair system plays an important role in maintaining genomic integrity and stability. To repair specific types of DNA damage and protect against carcinogenesis, human cells have evolved at least four repair pathways including base excision repair (BER). Poly(ADP-ribose) polymerase 1 (PARP1) functions as a key enzyme in the BER pathway. PARP1 consists of three domains: N-terminal DNA-binding domain, central automodification domain, and C-terminal catalytic domain (Cottet et al., 2000). In addition, the catalytic domain is divided into the N-terminal regulatory domain and C-terminal domain containing the active site (Ruf et al., 1998). PARP1 can detect and bind the damaged DNA by its DNA-binding domain, catalyze poly(ADP-ribosyl)-ation of target protein including itself using nicotinamide adenine dinucleotide (NAD+) as a substrate, recruit other DNA repair proteins to the damaged site, and eventually jointly perform DNA damage repair (Caldecott et al., 1996; El-Khamisy et al., 2003; Kim et al., 2005; Shiokawa et al., 2005). Besides DNA repair function, PARP1 is also implicated in other molecular and cellular processes such as chromatin modification, transcription and mitotic spindle formation (Kim et al., 2005). In recent years, the application of PARP inhibitors in patients with various cancers has improved patients’ clinical outcome, which highlights the crucial role of PAPR1 in tumorigenesis and progression.

Aberrant expression of *PARP1* has been recorded in different human cancers. *PARP1* expression level was significantly higher in colorectal and gastric cancer tissues than that in respective non-tumor tissues (Nosho et al., 2006; Liu et al., 2016). However, it was not the fact in a study on liver cancer, which demonstrated that non-cancerous liver tissues had a higher *PARP1* expression level than the liver cancer tissues (Krupa et al., 2017). In addition, promoter hypermethylation of PARP1, which might be associated with lower expression level of *PARP1*, predisposed females to breast cancer (Sabit et al., 2019). Therefore, PARP1 might play distinct roles in different tumors. Similarly, the results of animal experiments also supported the role of PARP1 in carcinogenesis. PARP1^-/- ^mice showed an increased risk of the lung, liver and colon cancer when treated by chemical carcinogens (Tsutsumi et al., 2001; Nozaki et al., 2003). Furthermore, *PARP1* expression level was associated with the survival of cancer patients (Gonçalves et al., 2011; Liu et al., 2016). For instance, gastric cancer patients with higher *PARP1 *expression level had significantly shorter overall survival and disease-free survival (Liu et al., 2016). Hence, PARP1 plays a part in tumorgenesis and progression.

Cixian of Hebei province is one of the high-incidence areas for esophageal cancer. The relative survival for esophageal cancer in Cixian had an upward trend from 2003 to 2013. However, the five-year relative survival for esophageal cancer remained low at 34.4% in 2013 (Li et al., 2018). Identifying applicable biomarkers for esophageal cancer prognosis may help to improve the outcome of esophageal cancer patients.

Accumulated evidences demonstrated that genetic polymorphisms in DNA repair genes may affect individual DNA repair capacity and change cancer risk (Hou et al., 2002; Wang et al., 2013). *PARP1* gene rs1136410 single nucleotide polymorphism (SNP) is a T to C transition at codon 762 located in the catalytic domain that leads to a change from valine to alanine, which is related to reduction of PARP1 enzymatic activity (Lockett et al., 2004; Wang et al., 2007). The rs8679 SNP is situated in the 3’-untranslated region (3’-UTR) of *PARP1 *gene, the T to C substitution may affect *PARP1* expression level (Teo et al., 2012; Schneiderova et al., 2017). These two polymorphisms were reported to be associated with risk of various tumors such as prostate cancer, esophageal cancer, cervical cancer, colorectal cancer, bladder cancer, and breast cancer (Hao et al., 2004; Lockett et al., 2004; Teo et al., 2012; Roszak et al., 2013; Schneiderova et al., 2017 ). In addition, some studies showed that these two polymorphisms might influence the prognosis of cancer patients (Kim et al., 2010; Zhou et al., 2015; Schneiderova et al., 2017). To date, no study has been conducted to assess whether *PARP1* rs1136410 and rs8679 SNPs, two potentially functional sites, are useful biomarkers to predict the survival of esophageal squamous cell carcinoma (ESCC) patients in Cixian high-incidence region.

## Materials and Methods

*Study subjects*

The survival information of 203 ESCC patients was collected. All the study subjects were ethnically homogeneous (of Han descent) and permanent residents of Cixian recruited during an endoscopic screening campaign between 2009 and 2014. The patients had histologically confirmed ESCC. Information on the sex, age, smoking habits and family history of upper gastrointestinal cancer (UGIC) from the cancer patients was obtained by two professional interviewers directly after blood sampling. Smokers were defined as those who formerly or currently smoked no less than five cigarettes per day for at least 2 years. Individuals who had at least one first-degree relative or at least two second-degree relatives who had esophageal/cardiac/gastric cancer were defined as having a family history of UGIC. The study was approved by the Ethics Committee of The Fourth Hospital of Hebei Medical University. The written informed consent forms were obtained from all recruited subjects.

*DNA extraction*

Five milliliters of venous blood was drawn from each subject in Vacutainer tubes containing ethylene diamine tetra acetic acid and stored at 4°C. After sampling, genomic DNA was extracted within 1 week by proteinase K (Merck, Darmstadt, Germany) digestion, followed by a salting out procedure according to the method published by Miller et al (Miller et al., 1988).

*Polymorphism genotyping*

The genotypes of *PARP1* gene polymorphisms were determined by the Shanghai Generay Biotech Co., Ltd. (Shanghai, China) using the polymerase chain reaction ligase detection reaction (PCR-LDR) method. The primers for amplification were 5’-ttctctgcatgtaggttttctctg-3’ and 5’-tgtaggccacctcgatgtc-3’ for rs1136410, 5’-ggaacgctaacaatttctcatac-3’ and 5’-gtcaagaatttcaaatgcaactt-3’ for rs8679, respectively. PCR reactions were carried out in a total volume of 15 µl including 50 ng genomic DNA, 1.5 µl 10× PCR buffer, 1.5 µl of 25 mM Mgcl2, 0.3 µl of 10 mM dNTPs, 0.25 µl of 10 pmol/µl each primer, and 1.25 U of Taq DNA-polymerase (TaKaRa, Japan). Cycling parameters were as follows: 94°C for 2 min; 35 cycles of 94°C for 15 s; 55°C for 15 s; 72°C for 25 s; and a final extension step at 72°C for 3 min. Three probes for LDR were synthesized for each SNP locus, which included two specific probes and one common probe. The two specific probes used to discriminate the specific bases were 5’-ctgttcttttgctcctccaggccaaggt-3’ and 5’-ttcttttgctcctccaggccaaggc-3’ for rs1136410, and 5’-ctgactgaaaagagctttccttctccaggaat-3’ and 5’-ctgaaaaagagctttccttctccaggaac-3’ for rs8679. The common probe was phosphorylated at the 5’ end and labeled at the 3’ end with 6-carboxy-fluorescein (FAM). For rs1136410 and rs8679, the common probes were 5’-P-ggaaatgcttgacaacctgctggac-FAM-3’ and 5’-P-actgaacatgggagctcttgaaatctga-FAM-3’ respectively. LDR reactions were performed in a 10µl reaction volume containing 3 µl of PCR product, 1 µl 10× Taq DNA ligase buffer, 0.01 µl of 10 pmol/µl each probe, 5 U Taq DNA ligase (New England Biolabs, USA). The LDR parameters were as follows: 25 cycles of 94°C for 30 s and 56°C for 1 min. After the LDR reaction, 1µl LDR reaction product was mixed with 10µl loading buffer, which contained marker. The mixture was then denatured at 95°C for 3 min, chilled immediately in ice water and analyzed on an ABI 3730XL DNA sequencer. In addition, the representative PCR products were subjected to direct DNA sequencing to confirm the accuracy of this method, with the results 100% concordant.

*Statistical analysis*

Statistical analysis was performed using the SPSS ver. 22.0 software package (SPSS, Chicago, IL, USA). P< 0.05 was considered significant for all statistical analyses. Survival time was calculated from the date of ESCC diagnosis to the date of death or last follow-up. The associations of survival time with demographic characteristics and *PARP1* gene SNPs were estimated using the Kaplan–Meier method and log-rank test. Univariate or multivariate Cox regression analysis was fitted to estimate the crude hazard ratios (HRs), adjusted HRs and 95% confidence intervals (CIs).

## Results

The mean age of the 203 ESCC patients was 60.4 ± 7.9 years. Sex, age, smoking status and UGIC family history were not associated with the survival time of the ESCC patients (Table 1).

**Figure1:**
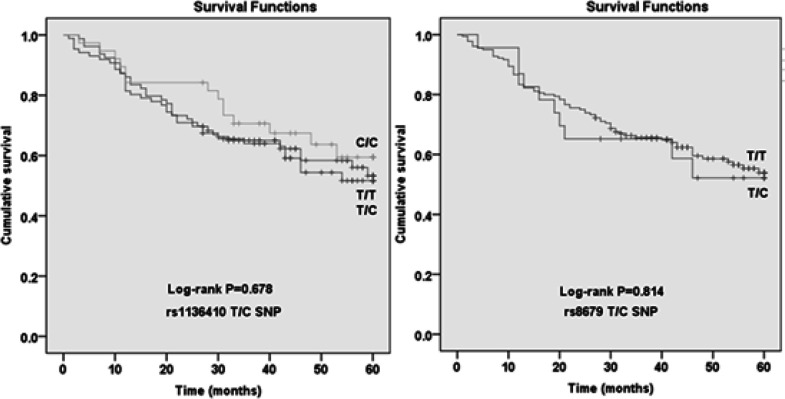
Kaplan-Merier survival curves for ESCC Patients by the Genotypes of *PARP1* Gene SNPs

The T/T, T/C and C/C genotype frequencies of rs1136410 in the ESCC patients were 38.9%, 42.4% and 18.7%, respectively. The mean survival time of rs1136410 T/T, T/C and C/C genotype carriers were 43.3, 42.3 and 46.6 months. Compared with the T/T genotype, the T/C genotype and C/C genotype did not affect the death risk of ESCC patients (HR= 1.159 and 0.823, 95%CI= 0.717-1.873 and 0.438-1.547) (Table 2, Figure 1). For rs8679, the T/T and T/C genotype frequencies of the ESCC patients were 88.7% and 11.3%. The mean survival time of T/T genotype carriers was 43.7 months, which was not significantly different from that of the patients with T/C genotype (P= 0.814). Compared with the T/T genotype, the T/C genotype did not modify the death risk of ESCC patients (HR= 1.130, 95%CI= 0.577-2.210) (Table 3, Fig. 1). When stratified by sex, age, smoking status and UGIC family history, rs1136410 and rs8679 SNPs were not associated with the survival time of ESCC patients (Table 2 and Table 3).

**Table 1:** ESCC Patients’ Characteristics and Survival of ESCC Patients

Group	Patients n (%)	Deaths n (%)^#^	MST* (months)	Log-rank P	HR (95% CI)
Sex					
Male	137 (67.5)	58 (42.3)	42.4		1
Female	66 (32.5)	25 (37.9)	46	0.473	0.843 (0.528~1.348)
Age					
≤60 years	103 (50.7)	42 (40.8)	43.9		1
>60 years	100 (49.3)	41 (41.0)	43.1	0.908	1.026 (0.667~1.577)
Smoking status					
Non-smoker	91 (44.8)	33 (36.3)	45.5		1
Smoker	112 (55.2)	50 (44.6)	42	0.279	1.272 (0.820~1.974)
Family history of UGIC					
Negative	128 (63.1)	58 (45.3)	41.8		1
Positive	75 (36.9)	25 (33.3)	46.4	0.127	0.697 (0.436~1.114)

^#^, The deaths’ number divided by the patients’ number in the row;*, mean survival time

**Table 2:** *PARP1 *Gene rs1136410 T/C SNP and Survival of ESCC Patients

SNP	Patients n (%)	Deaths n (%)^#^	MST (months)	Log-rank P	HR (95% CI)	HR (95% CI)*
Overall						
T/T	79 (38.9)	33 (41.8)	43.3		1	1
T/C	86 (42.4)	36 (41.9)	42.3		1.064 (0.663~1.708)	1.159 (0.717~1.873)
C/C	38 (18.7)	14 (36.8)	46.6	0.678	0.808 (0.433~1.511)	0.823 (0.438~1.547)
Male						
T/T	50 (36.5)	21 (42.0)	43.4		1	1
T/C	58 (42.3)	27 (46.6)	39.3		1.282 (0.723~2.272)	1.337 (0.749~2.386)
C/C	29 (21.2)	10 (34.5)	46.7	0.372	0.785 (0.370~1.669)	0.796 (0.372~1.702)
Female						
T/T	29 (43.9)	12 (41.4)	43.2		1	1
T/C	28 (42.4)	9 (32.1)	48.5		0.677 (0.285~1.609)	0.808 (0.334~1.958)
C/C	9 (13.7)	4 (44.4)	46.7	0.671	0.835 (0.267~2.613)	0.777 (0.240~2.514)
≤60 years						
T/T	36 (35.0)	14 (38.9)	45.3		1	1
T/C	45 (43.7)	20 (44.4)	41.4		1.293 (0.652~2.565)	1.304 (0.655~2.597)
C/C	22 (21.3)	8 (36.4)	46.5	0.655	0.942 (0.395~2.248)	0.828 (0.333~2.058)
>60 years						
T/T	43 (43.0)	19 (44.2)	41.7		1	1
T/C	41 (41.0)	16 (39.0)	43.5		0.997 (0.491~2.025)	0.876 (0.450~1.704)
C/C	16 (16.0)	6 (37.5)	46.3	0.791	0.873 (0.337~2.265)	0.736 (0.294~1.844)
Non-smoker						
T/T	35 (38.5)	14 (40.0)	44.3		1	1
T/C	45 (49.5)	16 (35.6)	45.1		0.927 (0.452~1.901)	1.029 (0.489~2.165)
C/C	11 (12.0)	3 (27.3)	50.2	0.673	0.573 (0.164~2.001)	0.576 (0.163~2.028)
Smoker						
T/T	44 (39.3)	19 (43.2)	42.5		1	1
T/C	41 (36.6)	20 (48.8)	39.3		1.235 (0.659~2.317)	1.277 (0.671~2.428)
C/C	27 (24.1)	11 (40.7)	45	0.635	0.885 (0.421~1.861)	0.852 (0.395~1.836)
Negative family history						
T/T	54 (42.2)	24 (44.4)	43		1	1
T/C	52 (40.6)	26 (50.0)	38.3		1.347 (0.772~2.349)	1.364 (0.779~2.385)
C/C	22 (17.2)	8 (63.4)	47.1	0.301	0.763 (0.343~1.700)	0.768 (0.344~1.718)
Positive family history						
T/T	25 (33.3)	9 (36.0)	44.2		1	1
T/C	34 (45.3)	10 (29.4)	48.4		0.724 (0.294~1.783)	0.880 (0.356~2.175)
C/C	16 (21.4)	6 (37.5)	45.9	0.766	0.920 (0.327~2.587)	0.809 (0.285~2.293)

^#^, The deaths’ number divided by the patients’ number in the row; *, Adjusted for sex, age, smoking status and UGIC family history

**Table 3:** *PARP1* Gene rs8679 T/C SNP and Survival of ESCC Patients

SNP	Patients n (%)	Deaths n (%)^#^	MST (months)	Log-rank P	HR (95% CI)	HR (95% CI)*
Overall						
T/T	180 (88.7)	73 (40.6)	43.7		1.000	1.000
T/C	23 (11.3)	10 (43.5)	42.1	0.814	1.082 (0.559~2.096)	1.130 (0.577~2.210)
Male						
T/T	125 (91.2)	51 (40.8)	43.0		1.000	1.000
T/C	12 (8.8)	7 (58.3)	35.6	0.268	1.552 (0.704~3.423)	1.577 (0.709~3.508)
Female						
T/T	55 (83.3)	22 (40.0)	45.3		1.000	1.000
T/C	11 (16.7)	3 (27.3)	49.1	0.482	0.652 (0.195~2.180)	0.723 (0.214~2.442)
≤60 years						
T/T	93 (90.3)	37 (39.8)	44.3		1.000	1.000
T/C	10 (9.7)	5 (50.0)	39.8	0.674	1.220 (0.479~3.106)	1.217 (0.471~3.143)
>60 years						
T/T	87 (87.0)	36 (41.4)	43.0		1.000	1.000
T/C	13 (13.0)	5 (38.5)	44.0	0.871	0.926 (0.363~2.359)	0.979 (0.378~2.535)
Non-smoker						
T/T	77 (84.6)	28 (36.4)	45.2		1.000	1.000
T/C	14 (15.4)	5 (35.7)	47.3	0.835	0.904 (0.349~2.342)	0.917 (0.353~2.381)
Smoker						
T/T	103 (92.0)	45 (43.7)	42.6		1.000	1.000
T/C	9 (8.0)	5 (55.6)	33.9	0.358	1.532 (0.607~3.866)	1.569 (0.614~4.011)
Negative family history						
T/T	114 (89.1)	52 (45.6)	41.6		1.000	1.000
T/C	14 (10.9)	6 (42.9)	43.7	0.803	0.899 (0.386~2.092)	0.892 (0.380~2.094)
Positive family history						
T/T	66 (88.0)	21 (31.8)	47.4		1.000	1.000
T/C	9 (12.0)	4 (44.4)	39.1	0.425	1.541 (0.528~4.498)	1.858 (0.606~5.697)

^#^, The deaths’ number divided by the patients’ number in the row; *, Adjusted for sex, age, smoking status and UGIC family history

## Discussion

In this study, we for the first time evaluated the association between *PARP1* rs1136410 and rs8679 SNPs and the survival of ESCC patients from Cixian high-incidence region. However, we found that these two polymorphisms might not be used as predictive biomarkers for the prognosis of these ESCC patients.

*PARP1* gene rs1136410 is a missense variant located in the catalytic domain. The loss of a methyl group from valine to alaline increases the distance between residue 762 in the regulatory domain and glycine 888, the closet neighbor of residue in the active site, looses the binding with NAD+ and reduces the catalytic activity (Cottet et al., 2000). The rs1136410 was documented to have an influence on risk of some tumors or prognosis of cancer patients (Hao et al., 2004; Lockett et al., 2004; Kim et al., 2010; Roszak et al., 2013; Zhou et al., 2015). On the contrary, Cottet et al did not find an association of rs1136410 with longevity-related difference in the poly(ADP-ribosyl)ation capacity (Cottet et al., 2000). Likewise, there was no significant relation between rs1136410 and PARP1 activity of 19 human cancer cell lines (Zaremba et al., 2009). Moreover, rs1136410 did not affect the level of beno[a]pyrene diol epoxide (BPDE)-induced DNA adducts (Yu et al., 2012). The difference of sample size and experimental method might contribute to the inconsistent results. Similar to some studies, we failed to find the association between rs1136410 and prognosis of ESCC patients (Gao et al., 2010; Li et al., 2013). Maybe, the discrepant role of PARP1 in cancer susceptibility or prognosis of cancer patients might be explained partly by PARP1’s involvement in various molecular and cellular processes.

*PARP1 *gene rs8679 SNP is located at microRNA-binding site, which might change the expression level of *PARP1 *by affecting the binding of microRNA with *PARP1* mRNA (Teo et al., 2012; Schneiderova et al., 2017). The rs8679 T/C genotype and C/C genotype were associated with increased risk of bladder cancer and breast cancer, respectively (Teo et al., 2012). In other studies, T/C or C/C genotype was related to decreased risk of colorectal cancer (Alhadheq et al., 2016; Schneiderova et al., 2017). Interestingly, rs8679 had no effect on susceptibility to neuroblastoma (Cheng et al., 2019). As for effect of rs8679 on prognosis of cancer patients, Cheng et al investigated the association of rs8679 with clinical outcome of colorectal cancer patients and found that C/C genotype carriers that received 5-FU-based chemotherapy had a shorter event-free survival (Schneiderova et al., 2017). In the previous study about bladder and present study on ESCC, no relation was observed between rs8679 and survival of cancer patients (Teo et al., 2012). To this day, limited studies have been conducted to test the possibility of rs8679 to be used as genetic biomarker for cancer susceptibility or prognosis of cancer patients. Therefore, it is necessary to determine the role of rs8679 in further studies on different tumors with larger sample size. Examining the association of *PARP1 *expression with different genotype of rs8679 in esophageal cancer tissues may be helpful in providing mechanistic evidence for the results.

There were some limitations in our study. Firstly, the sample size was relatively small, which might limit the statistical power. Secondly, only two potentially functional SNPs were involved in this study, we could not rule out the possibility of existing relation between other SNPs and ESCC patients’ survival. Thirdly, we failed to collect tumor grade, stage and treatment modalities, which might impact ESCC patients’ prognosis. The results of survival analyses would be more accurate if adjusted by the aforementioned factors. Fourthly, we did not measure the expression level of *PARP1* in esophageal cancer tissues with different genotype of rs1136410 and rs8679 SNPs. Therefore, our results should be interpreted cautiously.

In summary, the present study assessed the relation of *PARP1* rs1136410 and rs8679 SNPs with prognosis of ESCC patients from Cixian high-incidence region. The results indicated that these two SNPs might not be used as predictive markers for survival of ESCC patients. There is a need to explore whether other SNPs of *PARP1 *gene have an effect on prognosis of ESCC patients.
